# Environmental Gradients Explain Species Richness and Community Composition of Coastal Breeding Birds in the Baltic Sea

**DOI:** 10.1371/journal.pone.0118455

**Published:** 2015-02-25

**Authors:** Maria Nord, Pär Forslund

**Affiliations:** Department of Ecology, Swedish University of Agricultural Sciences, Uppsala, Sweden; Estación Biológica de Doñana, CSIC, SPAIN

## Abstract

Scientifically-based systematic conservation planning for reserve design requires knowledge of species richness patterns and how these are related to environmental gradients. In this study, we explore a large inventory of coastal breeding birds, in total 48 species, sampled in 4646 1 km^2^ squares which covered a large archipelago in the Baltic Sea on the east coast of Sweden. We analysed how species richness (α diversity) and community composition (β diversity) of two groups of coastal breeding birds (specialists, i.e. obligate coastal breeders; generalists, i.e. facultative coastal breeders) were affected by distance to open sea, land area, shoreline length and archipelago width. The total number of species per square increased with increasing shoreline length, but increasing land area counteracted this effect in specialists. The number of specialist bird species per square increased with decreasing distance to open sea, while the opposite was true for the generalists. Differences in community composition between squares were associated with differences in land area and distance to open sea, both when considering all species pooled and each group separately. Fourteen species were nationally red-listed, and showed similar relationships to the environmental gradients as did all species, specialists and generalists. We suggest that availability of suitable breeding habitats, and probably also proximity to feeding areas, explain much of the observed spatial distributions of coastal birds in this study. Our findings have important implications for systematic conservation planning of coastal breeding birds. In particular, we provide information on where coastal breeding birds occur and which environments they seem to prefer. Small land areas with long shorelines are highly valuable both in general and for red-listed species. Thus, such areas should be prioritized for protection against human disturbance and used by management in reserve selection.

## Introduction

Systematic conservation planning (SCP), aiming to preserve a variety of environments that can host a diversity of species [1], requires knowledge of how species are linked to the spatial variation of environmental conditions. Thereby, informed conservation measures like protection of areas can be implemented with regard to species richness and composition, or even to the protection of species of specific conservation concern [1]. Understanding how species richness (α diversity) and community composition (β diversity) are related to environmental variables and how they are distributed across the landscape is essential for successful conservation [2,3].

Coastal breeding birds (i.e. species confined to maritime habitats as well as species breeding in coastal estuaries and archipelagos) are of conservation concern, given that approximately 24% of all waterbird species are categorized as Globally Threatened in the IUCN Red List 2012 [4–6]. Such species (hereafter called specialist species since they are limited to coastal areas) may be especially vulnerable to increasing anthropogenic environmental pressures in the coastal landscape [4,7,8] as compared to other species that breed in this habitat but also in other habitats (hereafter called generalist species).

Studies of coastal breeding birds in archipelagos have generally focused on single or a few species (e.g., [9–12], but see [13]), and/or have focused on characteristics of breeding islands [9,10]. However, studies of large-scale patterns of α and β diversity of coastal breeding birds in relation to natural environmental gradients are lacking, and little is known of how coastal bird communities are distributed in the coastal landscapes of the Baltic Sea. Analysing such relationships is, however, fundamental for conducting SCP [1]. More specifically, the analysis needs to focus on how the regional species pool (γ diversity) is structured into the components α (within site) and β (between sites) diversity with regard to environmental gradients.

Beta diversity estimates are essential, since they give information on the spatial distribution of different species [14]. Science-based conservation planning can use β estimates to design a patchwork of different habitats that can host a variety of species and community types, rather than focusing simply on preserving high species richness [1,15]. Measuring β diversity is, however, not trivial [16,17,18]. In particular, large β estimates may be caused by real, ecological differences between sites (e.g. between squares in a grid) generating different community compositions, but may also be driven by differences in α diversity between those sites. In order to draw relevant conclusions from β diversity analysis about ecological processes generating spatial differences in community composition, it is necessary to account for the α-driven component of β estimates [17,19,20].

Our study area is a vast archipelago and the adjacent mainland coast in the Baltic Sea on the east coast of Sweden, consisting of more than 30 000 islands ranging from small, sparsely vegetated islets (<0.5 ha) to large, densely forested islands (up to 21000 ha) [21]. Increased exploitation (new houses, bridges, piers, quays etc.) close to the shoreline has reduced the amount of suitable breeding habitats and good foraging habitat for many birds [22,23]. In addition, boat tourism has increased in the last decades [24] which probably has increased the disturbance to birds. Population trends of coastal breeding birds in Sweden are reasonably well known; some populations have increased, e.g. those of some gulls and terns, whereas others have decreased, especially among waders and diving ducks [4]. Thus, from a conservation point of view, there is a need to investigate the diversity and distribution of coastal breeding birds, and specifically so for decreasing species and red-listed species.

Here, we analyse the coastal breeding bird fauna based on presence-absence data collected in nearly 4700 1 km^2^ squares, stretching approximately 240 km from north to south, and 50 km from east to west at its broadest extent. We classified birds breeding in this area as either coastal specialists (i.e., species breeding exclusively in the coastal landscape or with a disjunct distribution also in restricted parts of inland northern Sweden), or as coastal generalists (i.e., species breeding on the coast but also in inland wetlands) [25]. Since specialists are confined to the archipelago, conservation actions targeting coastal breeding birds should especially focus on this group. Another group potentially requiring specific attention is red-listed coastal breeders [26], of which several species were represented in our study. This group consists of both coastal specialists and generalists (seven of each). The overall aim of our study was to examine large-scale spatial patterns in species richness and community composition of coastal specialists and generalists and red-listed species in relation to environmental gradients. We specifically investigated the following questions: (1) how is species richness at the level of 1 km^2^ squares (α diversity) related to environmental gradients such as distance to open sea, archipelago width, shoreline length and land area, and, (2) how is community composition (β diversity) related to these environmental gradients. Our study covered a very large area and many sample units, which allowed for a large-scale analysis of diversity patterns and how they relate to environmental gradients. Finally, we discuss shortcomings of current conservation and management regimes and show how results from the current study can contribute to the implementation of a robust management plan within the framework of SCP.

## Methods

### Study area

**Fig 1:**
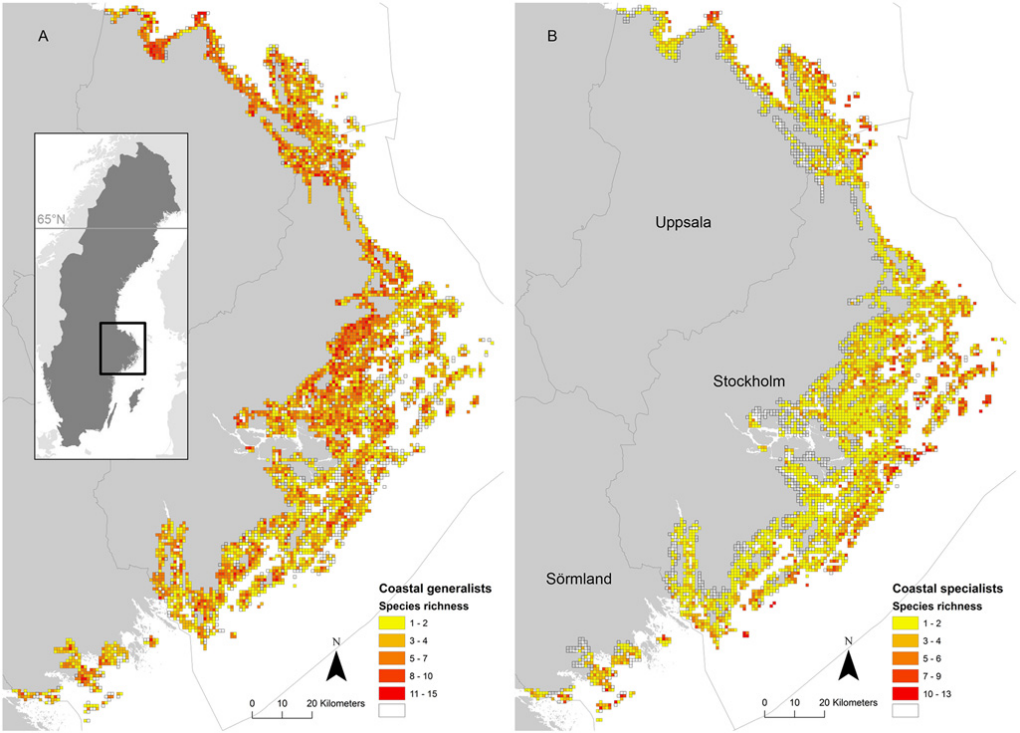
Species richness patterns of coastal specialist and generalist species. The archipelago is situated in the Baltic Sea off the east coast of Sweden. Observed number of species per square for (A) generalist species and (B) specialist species. The location of the counties are shown in (B).

The study area was situated in the Baltic Sea on the east coast of Sweden, and ranged from 58°36’N, 16°48’E in the south to 60°39’N, 17°33’E in the north (Fig. 1). The Baltic Sea is a non-tidal brackish sea (salinity varies between 3.5 and 7.0‰). The post-glacial land uplift in the archipelago region is approximately 5 mm/year, which has resulted in a general gradient where islands are larger closer to the mainland and smaller closer to the open sea. Thus, larger islands are generally less exposed to waves and winds, partially covered with forest and often contain agriculture and settlements. In the middle and outer zones, scattered groups of smaller islands are found that are more exposed and typically dominated by bare bedrock, heather moor, bushes and a few stunted trees or tree groups [21]. The archipelago is widest in the central part and narrows at the northern and southern limits (Fig. 1).

### Data collection

From 2000 to 2004, the county administrative boards of Stockholm, Södermanland and Uppsala performed inventories of coastal breeding bird species in the study area. Inventories of birds were primarily done with observations at distance so that birds were not disturbed. No specific permits were required. Permission to access land areas in bird protection areas during certain periods (mainly during June) was obtained from the county administrative board of each region. All necessary permits were obtained for the described study, which complied with all relevant regulations. The 48 species included in the survey were ducks, waders, gulls, auks and one passerine (Rock Pipit, *Anthus petrosus*), i.e. species that to some extent are dependent on shoreline habitat for breeding. The inventories were restricted to land within 100 m of the shoreline, and species records were summarised in each of the 1 km^2^ squares within the study area (Fig. 1). Squares were only included if they included both land and water, and had a minimum shoreline length of 25 m. This resulted in 4646 squares. Bird records from squares with a shoreline shorter than 25 m were assigned to the nearest squares fulfilling the criteria (n = 126). This had minimal influence on our results because the excluded squares were so few. Inventories were carried out on three occasions, in April, May and June, respectively, so that birds with different breeding phenology could be observed. Ducks were inventoried before and during the species’ normal egg laying period in the area when the breeding pair or the male is usually resting off the breeding island. Species breeding in early spring were inventoried in April and species breeding in late spring were inventoried in May. Great Crested Grebe *Podiceps cristatus* and Eurasian Coot *Fulica atra* were inventoried through nest observations during the time of nesting. Waders, gulls, auks and the Rock Pipit were inventoried in June during the time of their nesting or shortly after their young had hatched. The inventories were done by teams of two to three people on calm days without fog or rain. It was left to each inventory team to decide at what time of day they would perform the inventory, however the instructions stated that the early morning hours (04–12 am) should be preferred. Birds were assumed to be breeding, and hence the species recorded as present in the square, based on a combination of observations of parental behaviour, birds seen on nearby water (April and May) and nests found on land (June). A requirement for assumed breeding was that presence of the species was recorded in the relevant time period for the species (see above). On the first two occasions (April and May), the surveys were carried out entirely from open boats by cruising at low speeds around islands so that shores were easily surveyed. Time spent inventorying, i.e. survey effort, was determined by the number of birds, the observers ability to overview all sections of the shoreline and so forth, but was approximately 20–40 min/km^2^ during the first period (April) and usually 10–20 min/km^2^ during the second period (May). Speed of the boat and distance to the shore was adjusted so that birds were not disturbed (minimum distance to the shore approximately 25 meters; PF pers. obs.). This was done to avoid birds from flying around and thus making them difficult to count. On the third occasion (June) nest counts were conducted on land [27]. Detectability, observation errors and systematic differences between areas due to different teams of surveyors are factors that may cause individuals or species to be overlooked [28,29]. The design of the inventory, however, did not allow for accounting for these possible sources of error, although all field workers were skilled bird watchers using the same detailed inventory protocol.

In total, 19 species were classified as coastal specialists and 29 species as coastal generalists (for definitions, see Introduction). Fourteen of the species included were on the Swedish Red List [26]. There were seven red-listed species in each of the two groups; three specialists and two generalists are considered threatened (Vulnerable, VU) and the other species are classified as Near Threatened (NT) according to the present Red List of Sweden ([26]; Table 1).

**Table 1:** Occurrence frequencies and classifications of the studied coastal breeding bird species.

Species	Number of occurrences per square	Proportion of occupied squares (%)	Classification	Red-list status
Arctic Jaeger *Stercorarius parasiticus*	293	6.3	Specialist	
Arctic Tern *Sterna paradisaea*	1156	24.9	Specialist	
Barnacle Goose *Branta leucopsis*	121	2.6	Specialist	
Black Guillemot *Cepphus grylle*	218	4.7	Specialist	NT
Black-headed Gull *Larus ridibundus*	272	5.9	Generalist	
Black-throated Diver *Gavia arctica*	14	0.3	Generalist	
Canada Goose *Branta canadensis*	631	13.6	Generalist	
Caspian Tern *Hydroprogne caspia*	57	1.2	Specialist	VU
Common Eider *Somateria mollissima*	3163	68.1	Specialist	NT
Common Goldeneye *Bucephala clangula*	1596	34.4	Generalist	
Common Gull *Larus canus*	2453	52.8	Generalist	
Common Murre *Uria aalge*	14	0.3	Specialist	
Common Pochard *Aythya ferina*	56	1.2	Generalist	NT
Common Redshank *Tringa totanus*	491	10.6	Generalist	
Common Ringed Plover *Charadrius hiaticula*	132	2.8	Specialist	
Common Sandpiper *Actitis hypoleucus*	1091	23.5	Generalist	NT
Common Shelduck *Tadorna tadorna*	92	2.0	Specialist	
Common Teal *Anas crecca*	88	1.9	Generalist	
Common Tern *Sterna hirundo*	464	10.0	Generalist	
Eurasian Coot *Fulica atra*	405	8.7	Generalist	
Eurasian Curlew *Numenius arquata*	22	0.5	Generalist	VU
Eurasian Oystercatcher *Haematopus ostralegus*	1322	28.5	Specialist	
Eurasian Wigeon *Anas penelope*	13	0.3	Generalist	
Gadwall *Anas strepera*	100	2.2	Generalist	
Garganey *Anas querquedula*	9	0.2	Generalist	VU
Goosander *Mergus merganser*	2642	56.9	Generalist	
Great Black-backed Gull *Larus marinus*	1290	27.8	Specialist	
Great Cormorant *Phalacrocorax carbo sinensis*	38	0.8	Specialist	
Great Crested Grebe *Podiceps cristatus*	938	20.2	Generalist	
Greater Scaup *Aythya marila*	2	<0.1	Specialist	VU
Grey Heron *Ardea cinerea*	35	0.8	Generalist	
Greylag Goose *Anser anser*	933	20.1	Generalist	
Herring Gull *Larus argentatus*	721	15.5	Generalist	NT
Horned Grebe *Podiceps auritus*	25	0.5	Generalist	NT
Lesser Black-backed Gull *Larus fuscus*	342	7.4	Specialist	NT
Little Ringed Plover *Charadrius dubius*	2	<0.1	Generalist	
Mallard *Anas platyrhynchos*	2085	44.9	Generalist	
Mute Swan *Cygnus olor*	1964	42.3	Generalist	
Northern Lapwing *Vanellus vanellus*	79	1.7	Generalist	
Northern Pintail *Anas acuta*	9	0.2	Generalist	NT
Northern Shoveler *Anas clypeata*	90	1.9	Generalist	
Razorbill *Alca torda*	96	2.1	Specialist	
Red-breasted Merganser *Mergus serrator*	741	15.9	Specialist	
Red-necked Grebe *Podiceps grisegena*	2	<0.1	Generalist	
Rock Pipit *Anthus petrosus*	128	2.8	Specialist	
Ruddy Turnstone *Arenaria interpres*	313	6.7	Specialist	VU
Tufted Duck *Aythya fuligula*	978	21.1	Generalist	
Velvet Scoter *Melanitta fusca*	336	7.2	Specialist	NT

In total 48 coastal breeding bird species were inventoried in 4646 squares (1 × 1 km) in the archipelago situated in the Baltic Sea off the east coast of Sweden. NT = near threatened; VU = vulnerable, according to the national Red List of 2010 [26].

### Response variable calculation

Alpha (α) diversity was simply calculated as the total number of species, the number of specialist species, or the number of generalist species in each square. Beta (β) diversity, i.e. compositional dissimilarity, between squares was estimated by two different indices. For each pair of squares we calculated Sørensen’s dissimilarity index, given by β_S_ = 1–2*w*/(*a* + *b*) where *a* and *b* are the numbers of species in each plot of two samples, and *w* is the number of shared species [18]. This was done using the *dsvdis* function in the ‘labdsv’ R package [30]. This index does not account for differences in species numbers between pairs of squares. This may result in high β_S_ mainly due to the α-driven component of β_S_, i.e., the value of the index is dependent on the difference in numbers of species between the sites [19]. Thus, high β_S_ may not represent ‘true’ compositional dissimilarity in the sense of unique species in both sites [17]. To account for potentially confounding differences in the number of species per square, we also calculated the Raup-Crick estimate (β_RC_) of β diversity [17,19,31] by following the protocol of Chase et al. [19]. β_RC_ is a measure of the probability of finding the empirical value of the number of shared species compared to a null distribution of the number of shared species. The null distribution was generated by random draws of species in each square corresponding to the number of species observed in each of the two squares. The random draws were made from the regional species pool, weighted by the empirical probability of finding a species in a square in the region [19].

### Environmental variables

We investigated the effect of four variables, describing mainland and island characteristics, on species richness and composition: distance to open sea, archipelago width, shoreline length and total land area within the square. The choice of variables was motivated from our overall aim, which was to examine large-scale spatial patterns of diversity, but the variables have also previously been shown to correlate with the occurrence of coastal breeding birds in Finland, close to our study area [13]. Furthermore, according to general knowledge of the biology of coastal breeding birds, we assumed that the variables were relevant. To get estimates of the different environmental variables for each square, we used GIS layers that were obtained from the Topographical and General map, available through the Swedish Mapping, Cadastral and Land Registration Authority, and analysed using ArcMap 9.2 software (ArcGIS, ESRI, Redland, CA, USA). Distance to open sea was calculated by delimiting a 150 m wide buffer zone around the outermost islands (i.e. the islands closest to the open sea) and then measuring the Euclidean distance from the midpoint of each square to that buffer line. Archipelago width was estimated for each square by adding the Euclidean distance from the square’s midpoint to the nearest mainland to the distance to open sea. Shoreline length was the sum of all shorelines within each square, including islands and mainland. The total amount of land area in each square was calculated by subtracting the water surface area in each square from the total area of the square (1km^2^). Thus, the remaining area is the above water surface area.

### Statistical analysis

To determine how the number of coastal breeding bird species per square (α diversity) was related to environmental gradients, we modelled the number of species per square based on the four explanatory variables and all two-way interactions (with one exception, see below) using generalized linear mixed models (GLMMs). We used three response variables representing α diversity within each 1 km square: the total number of species per square, the number of coastal specialist species per square and the number of coastal generalist species per square. We also analysed red-listed species in total and within each group to see whether these species differed in their response to the environmental gradients. Prior to modelling we checked for correlations between the environmental variables and found mostly modest correlations (Pearson correlation for all pairwise combination of variables <0.35, except for distance to open sea vs. archipelago width which was 0.57). Thus, correlations were well below the criteria of Dormann et al. [32], stating that correlations <0.7 do not suffer severely from collinearity. We used a Gaussian probability distribution and used ln(number of species+1) as response variables (because there were some squares with zero species), with square identity as a random effect [33]. To improve model convergence, all explanatory variables were mean-standardised before analyses. To check for possible spatial autocorrelation in the data, we conducted tests of Moran’s *I* [34]. Since it is important to know whether spatial autocorrelation in the response variable remains after modelling the explanatory variables [34], Moran’s *I* was estimated from the residuals of preliminary GLMM models. Tests were conducted using the Variogram procedure in SAS 9.3 with normality assumption, binary weights and a lag distance of 1000 m, which is the distance between midpoints of adjacent squares. The tests suggested spatial autocorrelation of the residuals (specialist species: *I* = 0.157, p<0.0001; generalist species: *I* = 0.148, p<0.0001; all species: *I* = 0.162, p<0.0001; S1 Model Output). To account for spatial autocorrelation, we performed GLMM models including an error term assuming an exponential correlation structure by using the *glmmPQL* function in the ‘MASS’ package in R [35,36] (see S1 Model Output). For the model of generalists, including all two-way interactions caused convergence problems. We therefore removed the interaction between land area and archipelago width from this model, as initial analyses showed that this had minimal influence on the effects of the other terms in the model.

Because we wanted to examine the relationship between pairwise compositional dissimilarities (β_S_ and β_RC_) and pairwise differences in environmental variables, we used partial Mantel tests [37] which provide correlation estimates and tests for statistical significance [17]. In essence, the partial Mantel test investigates whether differences in species compositions between sites (measured with a β diversity index) are correlated with differences in characteristics (i.e. environmental variables) between the sites. Specifically, the Mantel test makes a nonparametric randomization test based on permutations of matrix elements since the elements of a distance matrix are not independent (all squares are compared with each other) [38,39]. The partial Mantel test specifically estimates the correlation coefficient while accounting for the other environmental variables [37]. In all Mantel tests, the environmental variables were expressed as distance matrices, which were derived as the distance, or difference, between the values of each pair of squares (i.e., the Manhattan method). The Mantel tests were performed using the ‘ecodist’ package in R [40]. Significance was determined at the level of p<0.05 and based on a total of 10 000 randomizations in each test.

## Results

The relative frequencies of species-specific occurrences in squares ranged from 68.1% (Common Eider) to <0.1% (Greater Scaup *Aythya marila*, Little Ringed Plover *Charadrius dubius*, and Red-necked Grebe *Podiceps grisegena*; Table 1). The most dominant species, occurring in more than 40% of the squares, were the Common Eider, Goosander *Mergus merganser*, Common Gull *Larus canus*, Mallard *Anas platyrhynchos*, and Mute Swan *Cygnus olor* (Table 1).

### Species richness (alpha diversity)

The total number of species found per square ranged between 0 and 25 (median = 5; 25% quartile = 3; 75% quartile = 8). The number of coastal specialist species found per square ranged between 0 and 13 (median = 2; quartiles = 1 and 3), and the number of coastal generalists found ranged between 0 and 15 (median = 3; quartiles = 2 and 6).

**Fig 2:**
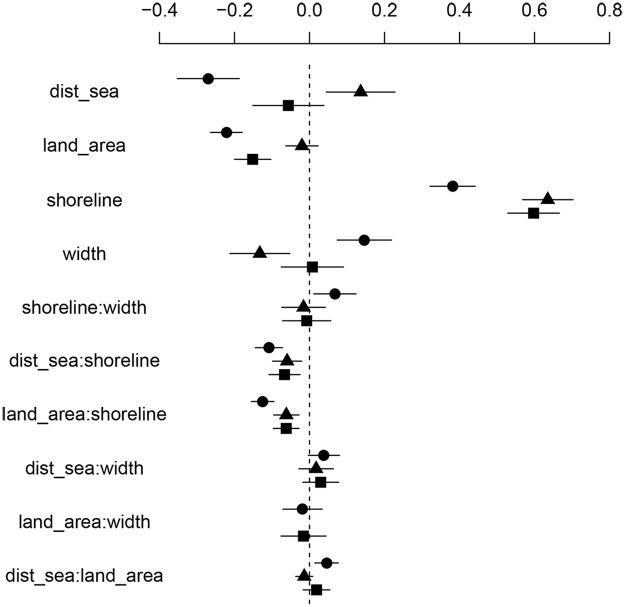
Parameter estimates and 95% confidence intervals for GLMMs for the different species groups. The number of specialist bird species per square (circles), the number of generalist bird species per square (triangles) and the total number of bird species per square (squares). The explanatory variables were the same in all three models, in the model for the generalists the interaction between land area and archipelago width was removed due to convergence problems. land_area = land area within each square; dist_sea = distance to open sea; shoreline = shoreline length; width = archipelago width. Interactions between variables are indicated by ‘:’.

**Fig 3:**
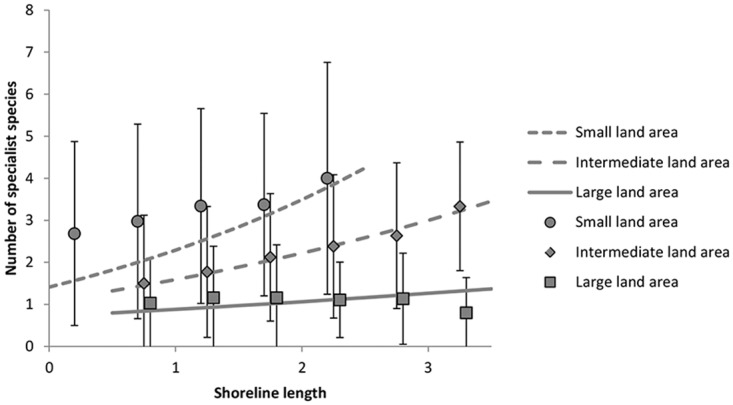
Interaction of shoreline length and land area on number of coastal specialist species per square. Lines show modelled interaction (see Fig. 2) for different levels of land area; data points (mean values ±1 standard deviation) show empirical estimates for land area ranges of 0–0.49, 0.5–0.99, 1–1.49 and so forth. Both shoreline length and land area were mean standardised.

**Fig 4:**
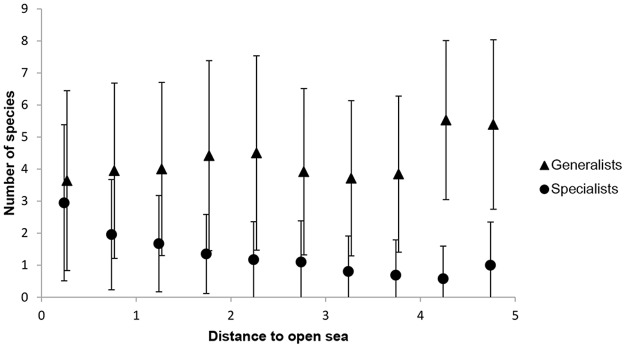
Mean number of species (± 1 standard deviation) in relation to distance to open sea. Data are grouped with regard to distance to open sea (mean standardised, x-axis value ±0.25, respectively). Sample sizes are (from left to right) 1879, 931, 683, 531, 247, 160, 112, 51, 19, 23.

The environmental variables (and interactions between them) had significant effects on the number of species in specialists (4 main effects and 4 interactions), generalists (3 main effects and 2 interactions) and all species combined (2 main effects and 2 interactions). Model outputs are given in S1 Model Output. The number of species per square increased with increasing shoreline length in both specialists (p<0.0001) and generalists (p<0.0001; Fig. 2). However, the effect of shoreline length was dependent on land area (interaction effect; Fig. 2); the number of specialist species increased strongly with increasing shoreline length when the land area was small, but the positive effect of shoreline decreased with increasing land area (p<0.0001; Fig. 3). Thus, the number of specialist species decreased with land area (negative main effect, p<0.0001; Fig. 2). In generalists, on the other hand, the shoreline effect on the number of species was generally positive (p<0.0001; Fig. 2) and only little influenced by land area (as revealed by a visual inspection of the interaction corresponding to Fig. 3, although the interaction effect was significant; p = 0.0003, Fig. 2). Distance to open sea had opposing effects on the two species groups; number of specialist species decreased with increasing distance to open sea (p<0.0001), whereas numbers of generalists increased (p = 0.004; Figs. 2 and 4). Archipelago width was negatively related to the number of generalist species (p = 0.001), but positively related to the number of specialists (p = 0.0001; Fig. 2), again showing a difference between the two groups. The different effects of distance to open sea, land area and archipelago width on specialists and generalists, respectively, indicate a turnover of species when moving along these gradients (i.e. from close to mainland to the open sea; see also the community composition analysis below).

The effect of the environmental variables on the number of red-listed species was similar to those for the entire species pool (S1 Fig., S1 Model Output). For specialist species, shoreline length (p<0.0001), land area (p<0.0001) and their interaction (p<0.0001) as well as distance to open sea (p = 0.0009) and archipelago width (p<0.0001) had similar effects (S1 Fig., S1 Model Output). For generalist species, the effect of archipelago width (p = 0.046) and shoreline length (p<0.0001) remained, whereas the effect of land area was supported (p = 0.007) and the effect of distance to open sea was reduced and not supported (p = 0.752). The interaction between land area and archipelago width was included in the model for the red-listed generalists, but was not supported (p = 0.076; S1 Fig., S1 Model Output). The total species pool of red-listed species were negatively influenced by land area (p<0.0001) and distance to open sea (p = 0.031), but positively affected by shoreline length (p<0.0001) and archipelago width (p<0.0001; S1 Fig., S1 Model Output).

### Spatial variation in community composition

The high values of the Sørensen’s dissimilarity index β_S_ indicated that the community composition varied considerably between squares (β_S_: mean ± SD = 0.71 ± 0.22 for all species, 0.73 ± 0.31 for coastal specialists and 0.73 ± 0.25 for coastal generalists). There was strong evidence that this variation in community composition between squares could be explained by all the environmental variables we investigated. This was revealed by partial Mantel tests, which showed that differences in community composition (measured as both β_S_ and β_RC_ of all species) between squares were significantly associated with differences in land area, distance to open sea, shoreline length and archipelago width, although the two latter had low correlations (Table 2). The differences in community composition along the environmental gradients were not just a consequence of different associations of specialist and generalist species, since similar correlations from partial Mantel tests within these groups were found, especially with regard to land area (Table 2). In addition, partial Mantel tests for red-listed species generally yielded similar results as those for the entire species pool (S1 Table). Differences in land area was still the most important factor for both red-listed specialists and generalists. However, measures of distance to open sea were not important for red-listed generalists (S1 Table).

**Table 2:** Correlations between differences in coastal breeding bird communities and differences in environmental variables.

Explanatory variables		All species		Specialist species		Generalist species	
		*r*	*P*	*r*	*P*	*r*	*P*
Distance to open sea	β_S_	0.091	<0.001	0.130	<0.001	0.027	<0.001
	β_RC_	0.094	<0.001	0.082	<0.001	0.060	<0.001
Land area	β_S_	0.249	<0.001	0.249	<0.001	0.130	<0.001
	β_RC_	0.197	<0.001	0.124	<0.001	0.115	<0.001
Shoreline length	β_S_	0.006	0.342	-0.050	<0.001	0.015	0.032
	β_RC_	0.035	<0.001	-0.028	<0.001	0.022	<0.001
Archipelago width	β_S_	-0.002	0.749	0.036	<0.001	-0.019	0.001
	β_RC_	0.016	<0.001	0.057	<0.001	-0.012	0.004

*r* values, estimated by partial Mantel tests, are the correlations of Sørensen’s dissimilarity index (β_S_) or the probabilistic Raup-Crick measure (β_RC_), respectively, with the distance matrices for the explanatory variables. *P* indicates statistical significance. Communities were defined as All species, Specialist species (i.e. bird species breeding only in the archipelago) or Generalist species (i.e. bird species breeding in the archipelago but also in inland lakes).

We found no clear indications that differences in the number of species between squares may have resulted in biased β_S,_ since correlation coefficients generally were not lower or higher when using β_S_ as compared to using β_RC_ (Table 2). Further, the two β diversity indices resulted in similar results from the partial Mantel tests with regard to which variables that best explained differences in community composition (Table 2).

## Discussion

Our analyses showed that the coastal breeding bird species found in the archipelago were unevenly distributed. Species richness changed along several environmental gradients, sometimes in opposite directions for specialists and generalists (e.g., in relation to distance to open sea). Furthermore environmental gradients sometimes interacted in their effects on species richness (e.g., the interaction between land area and shoreline length had a strong effect on the number of specialist species per square). As a result of the different distributions of specialists and generalists, we found that species turnover along the environmental gradients investigated resulted in differing species assemblages with regard to larger scales (e.g., distance to open sea) as well as smaller scales (e.g., land area and shoreline length). From a conservation point of view, the spatial structuring (e.g., the increasing number of specialist species closer to open sea) we found of the coastal bird community, especially for coastal specialists and red-listed species, is important when planning biodiversity protection measures. Such an analysis has not previously been performed on coastal breeding birds in the Baltic Sea.

### Species richness

The preference for breeding close to open sea among specialists was irrespective of archipelago width, which means that they also bred in areas where the archipelago is narrow and close to the mainland (Fig. 1B). Many of these species have been reported to also prefer islands with no or little vegetation [9–11,13], which are especially common in wind exposed localities close to the open sea [21]. Furthermore, these areas probably offer high quality foraging areas in the near vicinity and specialised feeders are probably dependent on breeding close to these foraging areas [10,41]. High predation risks due to the presence of mustelids and corvids closer to the mainland could be another reason for this preference of specialists for open sea [42]. Nordström & Korpimäki [43] suggested that many of these species moved further out towards the open sea when the American mink colonised islands closer to the mainland. However, if predation is a major factor affecting the distribution of specialists we should expect to find an absence of these species in narrow parts of the archipelago, but we clearly did not.

The observed preference of generalists to breed close to the mainland is similar to findings in the Finnish archipelago [13], and is in line with their general freshwater ecology. For example, the water is more brackish than further out in the archipelago and, thus, is more like mainland freshwater lakes [21]. Furthermore islands close to the mainland are often large with high vegetation.

As most coastal species bred close to water, shoreline length was positively related to the species richness of all coastal breeding birds, probably as a result of species-habitat area relationships [44,45]. By contrast, land area was negatively (specialist species) or not (generalist species) related to species richness in our study or appeared in an interaction effect with shoreline length. Hence, there were fewer specialist species in squares with large land areas, even with long shorelines (Fig. 3), suggesting that shoreline habitat was less suitable for specialist species in squares with larger land area than squares with smaller land area. Islands with a small land area may, in comparison to larger islands, be flatter, more open and have lower vegetation. Such features have been found to be attractive to coastal specialists in the nearby Finnish archipelago [10], and are highly likely to be similar in our study area. Rönkä et al. [10] found a positive relationship between island area and species richness, but this could be because they only inventoried small islands (<13.82 ha, mean area was 2.33 ha), where total land area nearly equals the area of suitable shore habitat.

### Community composition

Species turnover is usually stronger the steeper the environmental gradient is [14], and is often related to habitat heterogeneity [46]. Differences in community composition are often a consequence of spatial variation in habitat [46–49]. Since different species have different habitat preferences, habitat heterogeneity promotes and maintains spatial β diversity patterns [50–52]. Our analysis of β diversity indeed showed that the community changed along the gradients investigated, suggesting that the turnover of community composition was caused by changing ecological factors along the gradient and was not due to the α-driven component (i.e., that calculated differences in community composition may be caused by differences in species richness between squares [19]), as confirmed by the null model Raup-Crick β estimates. Since the relationships between species richness and some environmental gradients, especially distance to open sea, differed between specialists and generalists, species turnover was expected in the β diversity analysis of all species combined. However, there were differences in community composition with respect to the environmental variables also within specialists and generalists, which suggests species-specific habitat preferences within these groups as well.

Community composition was mainly driven by differences in land area and distance to open sea. Excluding the most common species, the Common Eider, did not change our results. Similar effects of land area and distance to open sea were found when we analysed red-listed specialist species (with and without the Common Eider) as well as red-listed generalist species. However, land area and distance to open sea are unlikely to be drivers of community composition, per se, but rather proxies for other factors that determine the presence of different species. For example, large land area in a square often indicates large islands, which differ from small islands in the extent of vegetation cover [10]. This, in turn, may affect the community composition, as suggested by studies from the nearby Finnish archipelago. For example, larids, Common Redshank *Tringa totanus* and Oystercatcher *Haematopus ostralegus* prefer open habitat and avoid breeding on islands with trees (i.e. large islands), presumably because trees favour crows which are important nest predators [10,13,41]. Other species, which are less sensitive to nest predation by crows, may benefit from the nesting habitats that large islands offer, such as suitable food and shelter from adverse weather [41]. Further, decreasing distance to open sea is generally related to increasing water depth [21], and this may affect the community composition. For example, species like Mallard, Oystercatcher, Common Ringed Plover *Charadrius hiaticula* and Common Redshank have been found to be associated with shallow waters, whereas large gulls and Black Guillemot *Cepphus grylle* were associated with deep water [10].

### Implications for conservation and management

The present management of coastal breeding birds depends on county administrative boards setting aside bird protection areas, where access to land and surrounding waters is prohibited during breeding. The motive for protection is to reduce presumed threats, i.e. human disturbance, in areas considered to be of conservation value for birds. Although such conservation measures may work, they are not founded on any clear, systematic approach to conservation with specified conservation targets [1]. For example, it is unclear whether the species present in the protected areas are representative of the community of coastal breeding bird species in the region, and whether this representation can be expected to be sustained in the long-term [1,53]. Further, there is no real analysis of the importance of these areas for the persistence of red-listed species needing specific conservation concern. The overall and long-term conservation values of the protected areas are therefore largely unknown. The current approach to achieve long-term conservation of biodiversity is Systematic Conservation Planning (SCP) [1]. SCP offers an operational framework that includes setting conservation goals, evaluating the current state of protection, compiling data on biodiversity, selecting new areas for protection (using some rationale), and implementing actions [1,53]. Our study makes important contributions to the SCP stages of data compilation and area selection. We have gathered data from a large region and connected the data with georeferenced information. Further, we have found important relationships between bird occurrence and environmental variables, which is fundamental information in the area selection process. For example, our study showed that community composition was associated with land area and distance to open sea. Assuming that an important conservation goal is to maintain a representation of the fauna of coastal breeding birds, a selection algorithm for designing a network of reserves would need to consider island size and location in the archipelago. Further variables to include should be shoreline length and distance to open sea, which were important predictors of species richness in our study.

Another and obvious conservation goal would be protection of red-listed species. Our very large study area enabled us to estimate the occurrence frequencies of those species, some of which were infrequently recorded (<60 out of 4646 squares for seven of the red-listed species; Table 1). We could link the presence of red-listed species to environmental gradients (S1 Model Output, S1 Fig., S1 Table), which is crucial information when selecting areas to protect.

Our results are useful when evaluating possible threats to the diversity of coastal breeding birds. In Sweden, the shoreline zone is generally protected through legislation. Any exemptions to the protection must be claimed and granted before the construction of new buildings etc. Further, grants must be based on evaluations of biodiversity values. Such information is often scarce, but here we provide information on where coastal breeding species occur and, even more important, which environments they seem to prefer. Hence, claims for exemptions should be evaluated in relation to, for example, unexploited long shorelines or closeness to open sea, which both are important factors for coastal specialists. Another threat is human disturbance, for example from increasing boat tourism. Our study showed that areas with small land area but long shorelines, presumably ‘mini-archipelagos’ of small islands, are highly valuable both in general and for red-listed species. Such areas should thus be prioritized for protection against human disturbance.

## Supporting Information

S1 Datafile (XLSX)

S1 Fig Parameter estimates and 95% confidence intervals for GLMMs for the red-listed species groups. The number of red-listed specialist bird species per square (circles), the number of red-listed generalist bird species per square (triangles) and the total number of red-listed bird species per square (squares). The explanatory variables were the same in all three models. land_area = land area within each square; dist_sea = distance to open sea; shoreline = shoreline length; width = archipelago width. Interactions between variables are indicated by ‘:’.
(TIF)

S1 Model Output Output from the GLMMs and Moran’s I test. (DOCX)

S1 Table Correlations between differences in red-listed coastal breeding bird communities and differences in environmental variables. *r* values, estimated by partial Mantel tests, are the correlation of Sørensen’s dissimilarity index with the distance matrices for the explanatory variables, as estimated by partial Mantel tests. *P* indicates statistical significance. Communities were defined as All red-listed species, Red-listed specialist species (i.e. red-listed bird species breeding only in the archipelago) or Red-listed generalist species (i.e. red-listed bird species breeding in the archipelago but also in inland lakes).
(DOCX)

## References

[1] Margules CR, Pressey RL. Systematic conservation planning. Nature. 2000;405: 243–253. 10821285 10.1038/35012251

[2] Arponen A, Moilanen A, Ferrier S. A successful community-level strategy for conservation prioritization. J Appl Ecol. 2008;45: 1436–1445.

[3] Ferrier S, Drielsma M. Synthesis of pattern and process in biodiversity conservation assessment: a flexible whole-landscape modelling framework. Divers Distrib. 2010;16: 386–402.

[4] Ottvall R, Edenius L, Elmberg J, Engström H, Green M, Holmqvist N, et al Population trends for Swedish breeding birds. Ornis Svecica. 2009;19: 117–192.

[5] Croxall JP, Butchart SHM, Lascelles B, Sattersfield AJ, Sullivan B, Symes A, et al Seabird conservation status, threats and priority actions: a global assessment. Bird Conserv Int. 2012;22: 1–34.

[6] Wetlands International. Waterbird Population Estimates, Fifth Edition Summary Report. Wetlands International, Wageningen, The Netherlands 2012.

[7] Butchart SHM, Walpole M, Collen B, van Strien A, Scharlemann JPW, Almond REA, et al Global biodiversity: indicators of recent declines. Science. 2010;328: 1164–1168. 10.1126/science.1187512 20430971

[8] Bader P, Edenius L, Lessmann J. Häckande kustfåglar på Holmöarna. Meddelande 13. Länsstyrelsen Västerbottens län. 2006 [in Swedish] 10.1371/journal.pone.0117162 25636148 PMC4455594

[9] Heinänen S, Rönkä M, von Numers M. Modelling the occurrence and abundance of a colonial species, the arctic tern *Sterna paradisaea* in the archipelago of SW Finland. Ecography. 2008;31: 601–611.

[10] Rönkä M, Tolvanen H, Lehikoinen E, von Numers M, Rautkari M. Breeding habitat preferences of 15 bird species on south-western Finnish archipelago coast: Applicability of digital spatial data archives to habitat assessment. Biol Conserv. 2008;141: 402–416.

[11] Heinänen S, von Numers M. Modelling species distribution in complex environments: an evaluation of predictive ability and reliability in five shorebird species. Divers Distrib. 2009;15: 266–279.

[12] Heinänen S, Erola J, von Numers M. High resolution species distribution models of two nesting water bird species: a study of transferability and predictive performance. Landsc Ecol. 2012;27: 545–555.

[13] von Numers M. Distribution, numbers and ecological gradients of birds breeding on small islands in the Archipelago Sea, SW Finland. Acta Zool Fenn. 1995;197: 1–127.

[14] Kessler M, Abrahamczyk S, Bos M, Buchori D, Dwi Putra D, Gradstein SR, et al Alpha and beta diversity of plants and animals along a tropical land-use gradient. Ecol Appl. 2009;19: 2142–2156. 20014584 10.1890/08-1074.1

[15] Anderson MJ, Tolimieri N, Millar RB. Beta diversity of demersal fish assemblages in the north-eastern pacific: Interactions of latitude and depth. PLOS ONE. 2013;8(3): e57918 10.1371/journal.pone.0057918 23526960 PMC3602450

[16] Tuomisto H. A diversity of beta diversities: straightening up a concept gone awry. Part 1. Defining beta diversity as a function of alpha and gamma diversity. Ecography. 2010;33: 2–22.

[17] Anderson MJ, Crist TO, Chase JM, Vellend M, Inouye BD, Freestone AL, et al Navigating the multiple meanings of β diversity: a roadmap for the practicing ecologist. Ecol Lett. 2011;14: 19–28. 10.1111/j.1461-0248.2010.01552.x 21070562

[18] Magurran AE, McGill BJ (eds.). Biological diversity: Frontiers in measurement and assessment. Oxford: Oxford University Press; 2011.

[19] Chase JM, Kraft NJB, Smith KG, Vellend M, Inouye BD. Using null models to disentangle variation in community dissimilarity from variation in α-diversity. Ecosphere. 2011;2: art24.

[20] Kraft NJB, Comita LS, Chase JM, Sanders NJ, Swenson NG, Crist TO, et al Disentangling the drivers of β diversity along latitudinal and elevational gradients. Science. 2011;333: 1755–1758. 10.1126/science.1208584 21940897

[21] Ås S, Bengtsson J, Ebenhard T. Archipelagos and theories of insularity. Ecological Bulletins. 1997;46: 88–116.

[22] Kindström M, Aneer G. What is happening to our shores? BALANCE Interim Report no. 26, Copenhagen, Denmark. 2006. Available: http://balance-eu.org.

[23] Aggemyr E, Cousins SAO. Landscape structure and land use history influence changes in island plant composition after 100 years. J Biogeogr. 2012;39: 1645–1656. 10.1007/s10295-012-1167-0 22842986

[24] Swedish Transport Agency. Båtlivsundersökningen 2010—en undersökning om svenska fritidsbåtar och hur de används . Sollentuna. 2010. [in Swedish]

[25] Svensson S, Svensson M, Tjernberg M. Svensk Fågelatlas. Vår Fågelvärld, supplement 31, Stockholm; 1999.

[26] Gärdenfors U (ed.). Rödlistade arter i Sverige 2010—The 2010 Red List of Swedish Species. ArtDatabanken, SLU, Uppsala; 2010 10.1016/j.jpba.2015.01.006 25636165

[27] Olsson L. Handledning för kustfågelinventerare i Stockholms län. Version 1.0. Sveriges Ornitologiska Förening. Kustfågelgruppen; 2000 [in Swedish] 10.1073/pnas.1216683110 23650377 PMC3666723

[28] Rosenstock SS, Anderson DR, Giesen KM, Leukering T, Carter MF. Landbird counting techniques: current practices and an alternative. Auk. 2002;119: 46–53.

[29] Aspenberg P. Metodjämförelse av undersökningstyper för kustfåglar. Rapport 2009: 12. Länsstyrelsen Gävleborg. 2009 [in Swedish] 10.1371/journal.pone.0117162 25636148 PMC4455594

[30] Roberts DW. labdsv: Ordination and Multivariate Analysis for Ecology. R package. 2010;1.4–1. Available: http://CRAN.R-project.org/web/packages/labdsv.

[31] Raup DM, Crick RE. Measurement of faunal similarity in paleontology. J Paleontol. 1979;53: 1213–1227.

[32] Dormann CF, Elith J, Bacher S, Buchmann C, Carl G, Carré G, et al Collinearity: a review of methods to deal with it and a simulation study evaluating their performance. Ecography. 2013;36: 27–46.

[33] Bolker BM, Brooks ME, Clark CJ, Geange SW, Poulsen JR, Stevens MHH, et al Generalized linear mixed models: a practical guide for ecology and evolution. Trends Ecol Evol. 2009;24: 127–35. 10.1016/j.tree.2008.10.008 19185386

[34] Beale CM, Lennon JJ, Yearsley JM, Brewer MJ, Elston DA. Regression analysis of spatial data. Ecol Lett. 2010;13: 246–264. 10.1111/j.1461-0248.2009.01422.x 20102373

[35] Ripley B, Venables B, Bates DM, Hornik K, Gebhardt A, Firth D. MASS . Support functions and datasets for Venables and Ripley’s MASS. R package. 2014;7 3–35. Available: http://cran.r-project.org/web/packages/MASS/MASS.pdf.

[36] R Development Core Team. R: A Language and Environment for Statistical Computing.—R Foundation for Statistical Computing. 2010 Available: http://www.R-project.org. 10.1371/journal.pone.0102642 25050811 PMC4106841

[37] Smouse PE, Long JC, Sokal RR. Multiple regression and correlation extensions of the Mantel test of matrix correspondence. Syst Zool. 1986;35: 627–632.

[38] Mantel N. The detection of disease clustering and a generalized regression approach. Cancer Res. 1967;27: 209–220. 6018555

[39] Sokal RR, Rohlf FJ. Biometry: the principles and practice of statistics in biological research. New York: W. H. Freeman and Company 1995.

[40] Goslee SC, Urban DL. The ecodist package for dissimilarity-based analysis of ecological data. J Stat Softw. 2007;22: 1–19.

[41] Hildén O. Habitat selection in birds. A review. Ann Zool Fennici. 1965;2: 53–75.

[42] Ahlén I, Andersson Å. Breeding ecology of an eider population on Spitsbergen. Ornis Scandinavica. 1970;1: 83–106.

[43] Nordström M, Korpimäki E. Effects of island isolation and feral mink removal on bird communities on small islands in the Baltic Sea. J Anim Ecol. 2004;73: 424–433.

[44] Arrhenius O. Species and area. J Ecol. 1921;9: 95–99.

[45] Gleason HA. On the relation between species and area. Ecology. 1922;3: 158–162.

[46] Veech JA, Crist TO. Habitat and climate heterogeneity maintain beta-diversity of birds among landscapes within ecoregions. Glob Ecol Biogeogr. 2007;16: 650–656.

[47] Brown JH, Mehlman DW, Stevens GC. Spatial variation in abundance. Ecology. 1995;76: 2028–2043. 8634995

[48] Mac Nally R, Fleishman E, Bulluck LP, Betrus CJ. Comparative influence of spatial scale on beta diversity within regional assemblages of birds and butterflies. J Biogeogr. 2004;31: 917–929.

[49] Reif J, Storch D, Símová I. The effect of scale-dependent habitat gradients on the structure of bird assemblages in the Czech Republic. Acta Ornithologica. 2008;43: 197–206.

[50] Loreau M. Biodiversity and ecosystem functioning: recent theoretical advances. Oikos. 2000;91: 3–17. 10927857

[51] Freestone AL, Inouye BD. Dispersal limitation and environmental heterogeneity shape scale-dependent diversity patterns in plant communities. Ecology. 2006;87: 2425–2432. 17089651 10.1890/0012-9658(2006)87[2425:dlaehs]2.0.co;2

[52] Suurkuukka H, Meissner KK, Muotka T. Species turnover in lake littorals: spatial and temporal variation of benthic macroinvertebrate diversity and community composition. Divers Distrib. 2012;18: 931–941.

[53] Kukkala AS, Moilanen A. Core concepts of spatial prioritisation in systematic conservation planning. Biol Rev. 2013;88: 443–464. 10.1111/brv.12008 23279291 PMC3654170

