# Supplementary figures and images for: Environmental Gradients Explain Species Richness and Community Composition of Coastal Breeding Birds in the Baltic Sea
Source: PLoS One. 2015 Feb 25;10(2):e0118455. doi: 10.1371/journal.pone.0118455 (PMC4340961; doi:10.1371/journal.pone.0118455)

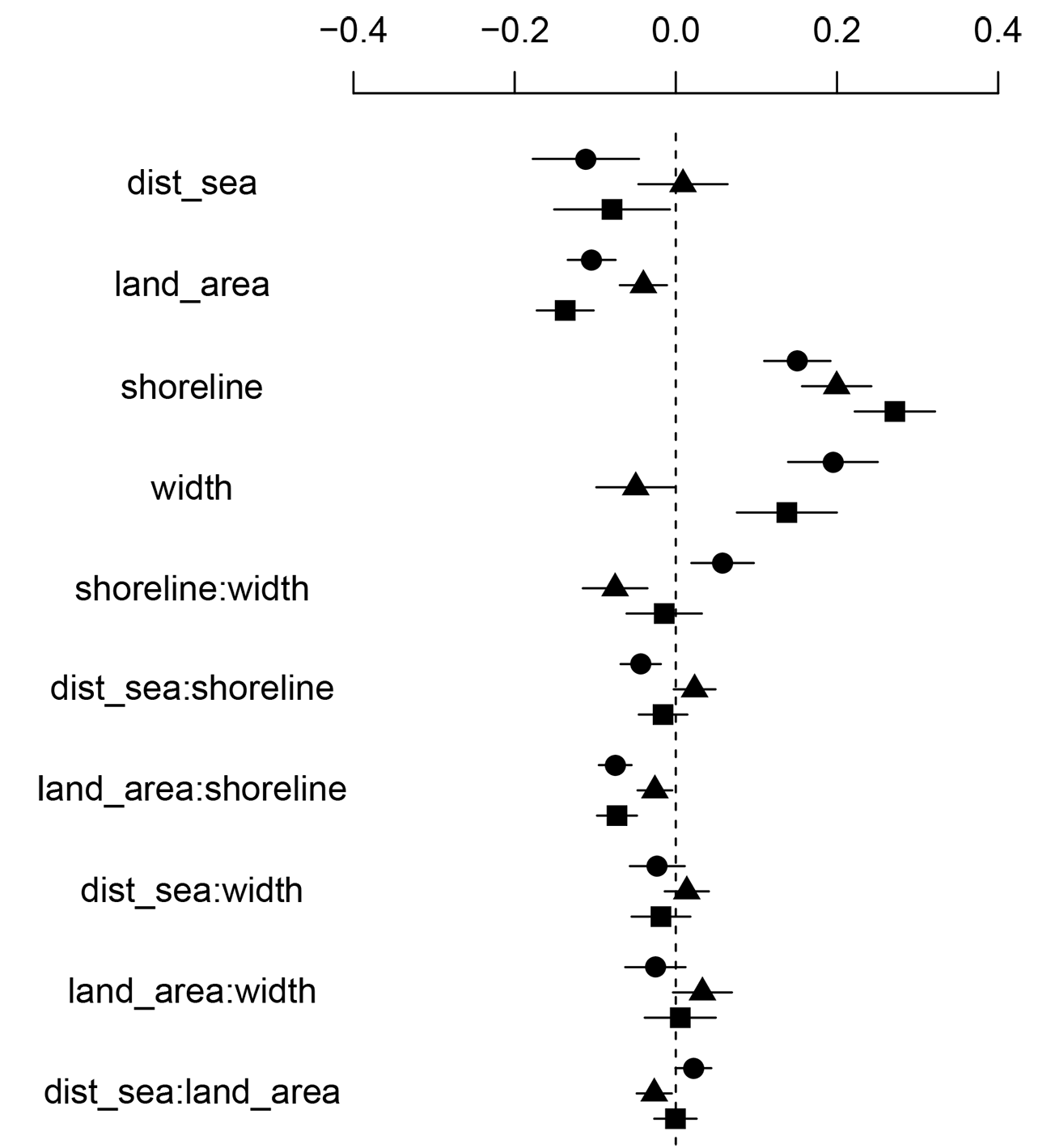

Supplement: S1 Fig — The number of red-listed specialist bird species per square (circles), the number of red-listed generalist bird species per square (triangles) and the total number of red-listed bird species per square (squares). The explanatory variables were the same in all three models. land_area = land area within each square; dist_sea = distance to open sea; shoreline = shoreline length; width = archipelago width. Interactions between variables are indicated by ‘:’. (TIF) [file pone.0118455.s002.tif]
