# Supplementary material for: Environmental Gradients Explain Species Richness and Community Composition of Coastal Breeding Birds in the Baltic Sea
Source: PLoS One. 2015 Feb 25;10(2):e0118455. doi: 10.1371/journal.pone.0118455 (PMC4340961; doi:10.1371/journal.pone.0118455)
Supplement: S1 Model Output — (DOCX) [file pone.0118455.s003.docx]

**Supporting Information: Summary of the GLMM models and Moran’s *I***

Content: (A) Summary of Generalized Linear Mixed Models fitted via PQL

(B) Summary of Moran’s *I*

**(A) Summary of GLMM models**

Explanatory variables

<name of expl.var in this document> = <name of expl.var in main paper>

dist_sea = Distance to open sea

land_area = Land area

shoreline = Shoreline length

width = Archipelago width

Formula used:

model <- glmmPQL(fixed = log<response+1> ~ dist_sea + land_area + shoreline + width + shoreline:width + shoreline:dist_sea + shoreline:land_area + width:dist_sea + width:land_area + dist_sea:land_area, random = ~1|square-ID, family = gaussian, control = list(lmeControl(returnObject=TRUE,opt=’optim’)))

struct.cor <- corSpatial(form=~long + lat, nugget=F, type= “exponential”, metric = “euclidean”)

struct.cor <- Initialize(struct.cor, data[,c(“long”,”lat”)])

model_SA <- update(model, correlation = struct.cor)

**Response variable: The number of total bird species per square**

AIC BIC logLik

NA NA NA

Random effects:

Formula: ~1 | ruta

(Intercept) Residual

StdDev: 0.5215737 0.4696027

Correlation structure: Exponential spatial correlation

Formula: ~long + lat | ruta

Parameter estimate(s):

range

637.663

Variance function:

Structure: fixed weights

Formula: ~invwt

Estimate Std. Error DF t-value p-value

(Intercept) 1.47748 0.04571 4635 32.32164 0.0000

dist_sea -0.05636 0.04825 4635 -1.16797 0.2429

land_area -0.15144 0.02472 4635 -6.12750 0.0000

shoreline 0.59748 0.03498 4635 17.08001 0.0000

width 0.00774 0.04231 4635 0.18295 0.8548

shoreline:width -0.00747 0.03273 4635 -0.22819 0.8195

dist_sea:shoreline -0.06661 0.02116 4635 -3.14755 0.0017

land_area:shoreline -0.06214 0.01748 4635 -3.55566 0.0004

dist_sea:width 0.02987 0.02437 4635 1.22601 0.2203

land_area:width -0.01612 0.03059 4635 -0.52675 0.5984

dist_sea:land_area 0.01883 0.01823 4635 1.03309 0.3016

Standardized within-group residuals:

Min Q1 Med Q3 Max

-2.32089 -0.38349 0.05544 0.45350 1.70542

**Response variable: The number of generalist bird species per square**

Please note that the interaction term between ‘land area’ and ‘archipelago width’ was excluded in this particular analysis.

AIC BIC logLik

NA NA NA

Random effects:

Formula: ~1 | ruta

(Intercept) Residual

StdDev: 0.355192 0.5230354

Correlation structure: Exponential spatial correlation

Formula: ~long + lat | ruta

Parameter estimate(s):

range

630.3574

Variance function:

Structure: fixed weights

Formula: ~invwt

Estimate Std. Error DF t-value p-value

(Intercept) 0.929658 0.044346 4636 20.9640 0.0000

dist_sea 0.136501 0.046721 4636 2.9216 0.0035

land_area -0.020092 0.022055 4636 -0.9110 0.3624

shoreline 0.635260 0.034301 4636 18.5204 0.0000

width -0.132380 0.040598 4636 -3.2608 0.0011

shoreline:width -0.016048 0.029561 4636 -0.5429 0.5872

dist_sea:shoreline -0.059677 0.019855 4636 -3.0056 0.0027

land_area:shoreline -0.061792 0.017270 4636 -3.5780 0.0003

dist_sea:width 0.017695 0.023474 4636 0.7538 0.4510

dist_sea:land_area -0.014014 0.011522 4636 -1.2163 0.2239

Standardized within-group residuals:

Min Q1 Med Q3 Max

-3.1442184 -0.5016198 0.1065490 0.6076078 2.1448106

**Response variable: The number of specialist bird species per square**

AIC BIC logLik

NA NA NA

Random effects:

Formula: ~1 | ruta

(Intercept) Residual

StdDev: 0.3488356 0.4539839

Correlation structure: Exponential spatial correlation

Formula: ~long + lat | ruta

Parameter estimate(s):

range

642.8602

Variance function:

Structure: fixed weights

Formula: ~invwt

Estimate Std. Error DF t-value p-value

(Intercept) 1.01547 0.03988 4635 25.4659 0.0000

dist_sea -0.26970 0.04208 4635 -6.4093 0.0000

land_area -0.22148 0.02147 4635 -10.3141 0.0000

shoreline 0.38178 0.03071 4635 12.4328 0.0000

width 0.14637 0.03681 4635 3.9760 0.0001

shoreline:width 0.06775 0.02872 4635 2.3587 0.0184

dist_sea:shoreline -0.10846 0.01857 4635 -5.8417 0.0000

land_area:shoreline -0.12471 0.01532 4635 -8.1406 0.0000

dist_sea:width 0.03844 0.02118 4635 1.8147 0.0696

land_area:width -0.01861 0.02673 4635 -0.6962 0.4863

dist_sea:land_area 0.04554 0.01594 4635 2.8576 0.0043

Standardized within-group residuals:

Min Q1 Med Q3 Max

-2.498044044 -0.562345588 -0.006921773 0.568245737 2.241613196

**Response variable: The number of total red-listed bird species per square**

AIC BIC logLik

NA NA NA

Random effects:

Formula: ~1 | ruta

(Intercept) Residual

StdDev: 0.3138132 0.3778644

Correlation Structure: Exponential spatial correlation

Formula: ~long + lat | ruta

Parameter estimate(s):

range

693.5045

Variance function:

Structure: fixed weights

Formula: ~invwt

Value Std.Error DF t-value p-value

(Intercept) 0.6965629 0.03425658 4635 20.333699 0.0000

dist_sea -0.0791792 0.03660218 4635 -2.163238 0.0306

land_area -0.1372527 0.01798757 4635 -7.630421 0.0000

shoreline 0.2718454 0.02542481 4635 10.692130 0.0000

width 0.1378096 0.03164942 4635 4.354252 0.0000

shoreline:width -0.0143952 0.02383953 4635 -0.603838 0.5460

dist_sea:shoreline -0.0158341 0.01539516 4635 -1.028510 0.3038

land_area:shoreline -0.0729765 0.01263875 4635 -5.774032 0.0000

dist_sea:width -0.0184217 0.01860233 4635 -0.990288 0.3221

land_area:width 0.0055132 0.02246828 4635 0.245378 0.8062

dist_sea:land_area -0.0004890 0.01339016 4635 -0.036519 0.9709

Standardized Within-Group Residuals:

Min Q1 Med Q3 Max

-2.15748276 -0.50765884 -0.05793719 0.53259104 2.80547563

**Response variable: The number of red-listed generalist bird species per square**

AIC BIC logLik

NA NA NA

Random effects:

Formula: ~1 | ruta

(Intercept) Residual

StdDev: 8.781232e-05 0.3580589

Correlation Structure: Exponential spatial correlation

Formula: ~long + lat | ruta

Parameter estimate(s):

range

591.7286

Variance function:

Structure: fixed weights

Formula: ~invwt

Value Std.Error DF t-value p-value

(Intercept) 0.22228944 0.02714831 4635 8.187967 0.0000

dist_sea 0.00890759 0.02821704 4635 0.315681 0.7523

land_area -0.04023855 0.01491640 4635 -2.697605 0.0070

shoreline 0.19962320 0.02192943 4635 9.102982 0.0000

width -0.04972578 0.02495826 4635 -1.992358 0.0464

shoreline:width -0.07531411 0.02044206 4635 -3.684273 0.0002

dist_sea:shoreline 0.02334059 0.01322692 4635 1.764628 0.0777

land_area:shoreline -0.02596701 0.01096586 4635 -2.367986 0.0179

dist_sea:width 0.01361806 0.01397719 4635 0.974306 0.3300

land_area:width 0.03304086 0.01863385 4635 1.773163 0.0763

dist_sea:land_area -0.02674407 0.01112636 4635 -2.403667 0.0163

Standardized Within-Group Residuals:

Min Q1 Med Q3 Max

-1.6730358 -0.7696370 -0.5260292 1.0208702 3.4315400

**Response variable: The number of red-listed specialist bird species per square**

AIC BIC logLik

NA NA NA

Random effects:

Formula: ~1 | ruta

(Intercept) Residual

StdDev: 0.2626091 0.3292649

Correlation Structure: Exponential spatial correlation

Formula: ~long + lat | ruta

Parameter estimate(s):

range

810.012

Variance function:

Structure: fixed weights

Formula: ~invwt

Value Std.Error DF t-value p-value

(Intercept) 0.5567144 0.03068946 4635 18.140248 0.0000

dist_sea -0.1116371 0.03360765 4635 -3.321775 0.0009

land_area -0.1044706 0.01513156 4635 -6.904154 0.0000

shoreline 0.1507924 0.02098960 4635 7.184148 0.0000

width 0.1949841 0.02835316 4635 6.876977 0.0000

shoreline:width 0.0580519 0.01976291 4635 2.937416 0.0033

dist_sea:shoreline -0.0435694 0.01273824 4635 -3.420359 0.0006

land_area:shoreline -0.0751909 0.01035602 4635 -7.260596 0.0000

dist_sea:width -0.0229701 0.01736073 4635 -1.323110 0.1859

land_area:width -0.0253451 0.01906762 4635 -1.329223 0.1838

dist_sea:land_area 0.0217714 0.01135862 4635 1.916731 0.0553

Standardized Within-Group Residuals:

Min Q1 Med Q3 Max

-2.56252119 -0.54741784 0.01989464 0.52059146 2.63230147

**(B) Table of Moran’s *I***

| Response variable | Observed (*I*) | Expected (I) | Std Dev | Z | *p*-value |
| --- | --- | --- | --- | --- | --- |
| The number of total bird species per square | 0.162 | -0.000217 | 0.0118 | 13.8 | <.0001 |
| The number of generalist bird species per square | 0.148 | -0.000217 | 0.0118 | 12.6 | <.0001 |
| The number of specialist bird species per square | 0.157 | -0.000217 | 0.0117 | 13.4 | <.0001 |
