# Supplementary material for: Environmental Gradients Explain Species Richness and Community Composition of Coastal Breeding Birds in the Baltic Sea
Source: PLoS One. 2015 Feb 25;10(2):e0118455. doi: 10.1371/journal.pone.0118455 (PMC4340961; doi:10.1371/journal.pone.0118455)
Supplement: S1 Table — r values, estimated by partial Mantel tests, are the correlation of Sørensen’s dissimilarity index with the distance matrices for the explanatory variables, as estimated by partial Mantel tests. P indicates statistical significance. Communities were defined as All red-listed species, Red-listed specialist species (i.e. red-listed bird species breeding only in the archipelago) or Red-listed generalist species (i.e. red-listed bird species breeding in the archipelago but also in inland lakes). (DOCX) [file pone.0118455.s004.docx]

**Table S1.** **Correlations between differences in red-listed coastal breeding bird communities and differences in environmental variables.** *r* values, estimated by partial Mantel tests, are the correlation of Sørensen’s dissimilarity index with the distance matrices for the explanatory variables, as estimated by partial Mantel tests. *P* indicates statistical significance. Communities were defined as All red-listed species, Red-listed specialist species (i.e. red-listed bird species breeding only in the archipelago) or Red-listed generalist species (i.e. red-listed bird species breeding in the archipelago but also in inland lakes).

| **Variables** | **All red-listed species** | | **Red-listed specialist species** | | **Red-listed generalist species** | |
| --- | --- | --- | --- | --- | --- | --- |
|  | ***r*** | ***P*** | ***r*** | ***P*** | ***r*** | ***P*** |
| **Distance to open sea** | 0.109 | <0.001 | 0.111 | <0.001 | 0.012 | 0.047 |
| **Land area** | 0.254 | <0.001 | 0.252 | <0.001 | 0.076 | <0.001 |
| **Shoreline length** | -0.036 | <0.001 | -0.055 | <0.001 | 0.016 | 0.001 |
| **Archipelago width** | 0.042 | <0.001 | 0.048 | <0.001 | -0.018 | <0.001 |
